# Uncovering new disease indications for G-protein coupled receptors and their endogenous ligands

**DOI:** 10.1186/s12859-018-2392-y

**Published:** 2018-10-01

**Authors:** Johannes M Freudenberg, Ian Dunham, Philippe Sanseau, Deepak K Rajpal

**Affiliations:** 1 Computational Biology, Target Sciences, GlaxoSmithKline, Collegeville, PA 19426 USA; 2 Open Targets, Wellcome Genome Campus, Hinxton, Cambridge, CB10 1SD UK; 3 European Molecular Biology Laboratory, European Bioinformatics Institute (EMBL-EBI), Wellcome Genome Campus, Hinxton, Cambridge, CB10 1SD UK; 4 Computational Biology and Stats, Target Sciences, GSK Medicines Research Centre, Gunnels Wood Road, Stevenage, SG1 2NY UK

**Keywords:** G-protein coupled receptors, Drug discovery, Data integration, Target identification

## Abstract

**Background:**

The Open Targets Platform integrates different data sources in order to facilitate identification of potential therapeutic drug targets to treat human diseases. It currently provides evidence for nearly 2.6 million potential target-disease pairs. G-protein coupled receptors are a drug target class of high interest because of the number of successful drugs being developed against them over many years. Here we describe a systematic approach utilizing the Open Targets Platform data to uncover and prioritize potential new disease indications for the G-protein coupled receptors and their ligands.

**Results:**

Utilizing the data available in the Open Targets platform, potential G-protein coupled receptor and endogenous ligand disease association pairs were systematically identified. Intriguing examples such as GPR35 for inflammatory bowel disease and CXCR4 for viral infection are used as illustrations of how a systematic approach can aid in the prioritization of interesting drug discovery hypotheses. Combining evidences for G-protein coupled receptors and their corresponding endogenous peptidergic ligands increases confidence and provides supportive evidence for potential new target-disease hypotheses. Comparing such hypotheses to the global pharma drug discovery pipeline to validate the approach showed that more than 93% of G-protein coupled receptor-disease pairs with a high overall Open Targets score involved receptors with an existing drug discovery program.

**Conclusions:**

The Open Targets gene-disease score can be used to prioritize potential G-protein coupled receptors-indication hypotheses. In addition, availability of multiple different evidence types markedly increases confidence as does combining evidence from known receptor-ligand pairs. Comparing the top-ranked hypotheses to the current global pharma pipeline serves validation of our approach and identifies and prioritizes new therapeutic opportunities.

**Electronic supplementary material:**

The online version of this article (10.1186/s12859-018-2392-y) contains supplementary material, which is available to authorized users.

## Background

There are currently 827 known human G-protein coupled receptors (GPCRs) of which 406 are non-olfactory [1]. Together, this amounts to approximately 2% of all known protein-coding genes. They are, however, the largest ‘target’ class of the ‘druggable genome’ representing approximately 19% of the currently available drug targets [2, 3]. They have long played a prominent role in drug discovery [4] – so much so, that as of this writing, 475 FDA approved drugs act on GPCRs [5]. Several reasons account for this over-representation. GPCRs have ligand binding sites on the outer cell surface membrane, and potent effects can be achieved even from small ligand concentrations [2]. Some, but not all GPCRs have endogenous peptidergic ligands, small proteins produced by other cells that bind to the GPCR and trigger the downstream signalling cascade. Thus, endogenous peptides also provide a good starting point for the design of potential new drug targets due to their high tractability, specificity, safety, tolerability, and efficacy, as well as lower production complexity than other biopharmaceuticals [6]. These characteristics make GPCRs and their endogenous peptidergic ligands an extremely promising category of drug targets to investigate [7, 8].

To link potential drug targets, such as GPCRs, to disease indications, several public databases integrating various types of evidence are available including PHAROS [9], DisGeNET [10], The Monarch Initiative [11], and DISEASES [12] as well as the recently developed Open Targets platform [13]. This public-private platform integrates a large number of different data sources to provide evidence supporting the association between genes which could be known or new potential drug targets and human diseases [13]. As of October 2017, the Open Targets platform covers more than 26,000 genes which include both protein-coding as well non-coding gene identifiers and 9150 disease and phenotypic terms. In total, it consolidates evidence for nearly 2.6 million potential target-disease pairs. A scoring scheme was developed capturing the overall confidence and strength of a target-disease association given the available evidence such that the resulting association score ranging from 0 (“no evidence”) to 1 (“strongest evidence”) combines the observation frequency, the magnitude or strength, and the confidence in the source of evidence for a given target-disease association [13].

This is an exceedingly large number of hypotheses to analyse which raises the question of how a drug discovery scientist might prioritize amongst them. Potential ranking strategies might include the overall Open Targets score, the number of different types of evidence supporting the hypothesis, or other measures computed over the Open Targets database, such as mutual information [14] or machine learning approaches [15], that relate a given target-disease pair to other, similar hypotheses. Criteria to consider that are highly relevant to drug discovery but may currently reside outside the scope of the Open Targets platform include, for example, disease incidence and prevalence, unmet medical need, the availability of disease models and biomarkers [16], and druggability of the target [17].

Here we hypothesize that the evidence collected in the Open Targets platform supporting gene-disease associations can be used effectively to identify and prioritize target hypotheses for drug discovery. Focussing on a protein class of particular interest to drug discovery as a use case, we outline an innovative approach to identify and prioritize potential new GPCR and endogenous peptidergic therapeutic targets using the data behind the target-disease pairs from the Open Targets platform. First, we describe the distribution of the target-disease pairs and corresponding scores in the Open Targets platform database. Then we identify and characterize sets of GPCRs as well as their endogenous peptidergic ligands in the context of the Open Targets platform. Lastly, we compare the top-ranked GPCR and peptidergic targets to the current global pharma pipeline to validate our approach and to identify potential new disease indications and therapeutic opportunities.

## Results

### Distribution of the overall Open Targets score and relationship to individual data types

**Fig. 1:**
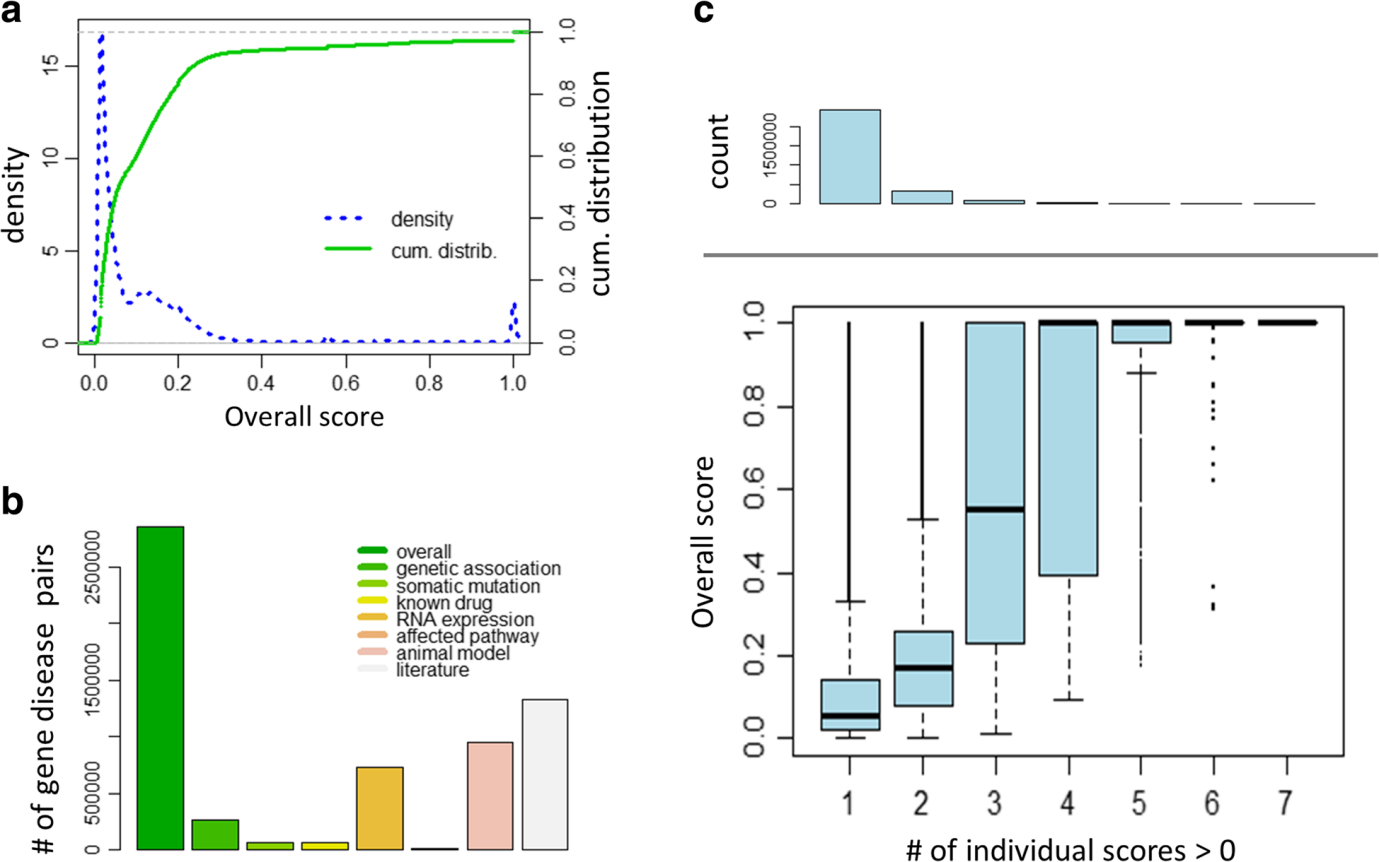
Distribution of the overall Open Targets score and relationship to individual data types. **a** Empirical density and cumulative distribution of the Open Targets score. Density and distribution functions were estimated using the R functions *density()* and *ecdf()*, respectively, with default parameters and using all pairs and 10,000 randomly selected pairs, respectively. **b** Number of gene-disease pairs with positive scores by type of score (overall, genetic association, somatic mutation, known drugs, RNA expression, affected pathways, animal models, and literature mining). **c** Comparison of the overall score of a disease-gene association and the number of data sources where the individual data type score is > 0. The top panel shows the counts of target-disease pairs corresponding to the scores below

At the time of this analysis the Open Targets Platform integrates fifteen different data sources organized into seven different data types: genetic association, somatic mutations, RNA expression, known drug targets, affected molecular pathways, animal models, and text mining [13]. Each gene-disease pair receives a set of scores, each ranging from 0 to 1, representing the seven different data types as well as an overall cumulative score. These scores are designed to incorporate measures of the frequency, effect size, and confidence of the observed gene-disease evidence [13]. To examine the distribution of the resulting scores we plotted the empirical density and the cumulative distribution of the overall score, respectively (Fig. 1a). These plots suggest a mixture of distributions with most gene-disease pairs receiving scores near zero (median overall score = 0.057), a relatively broad peak around 0.15 and two other peaks around 0.55 and 1, respectively. Interestingly, the 95th percentile of the overall score is approximately 0.5 and approximately 2.7% of the gene-disease pairs in Open Targets have the maximum score of 1.

As has been observed elsewhere [18], the number of gene-disease pairs with a positive score varies considerably between the different data types. For example, 46% of the pairs had a literature mining score greater than zero compared to less than 5% of pairs with somatic mutation, known drugs, or affected pathways scores greater than zero, respectively (Fig. 1b). While the Open Targets Platform integrates many different data sources, most disease-gene associations (97%) are supported by only one or two different data sources and only a fraction of pairs (0.44%) have evidence from 4 or more different types of data sources. Comparing the overall score of a disease-gene association against the number of data types where the individual data type score is positive shows that the more independent data sources supporting a given gene-disease association the higher the overall score (Fig. 1c).

### Characterizing GPCRs and endogenous ligands

**Fig. 2:**
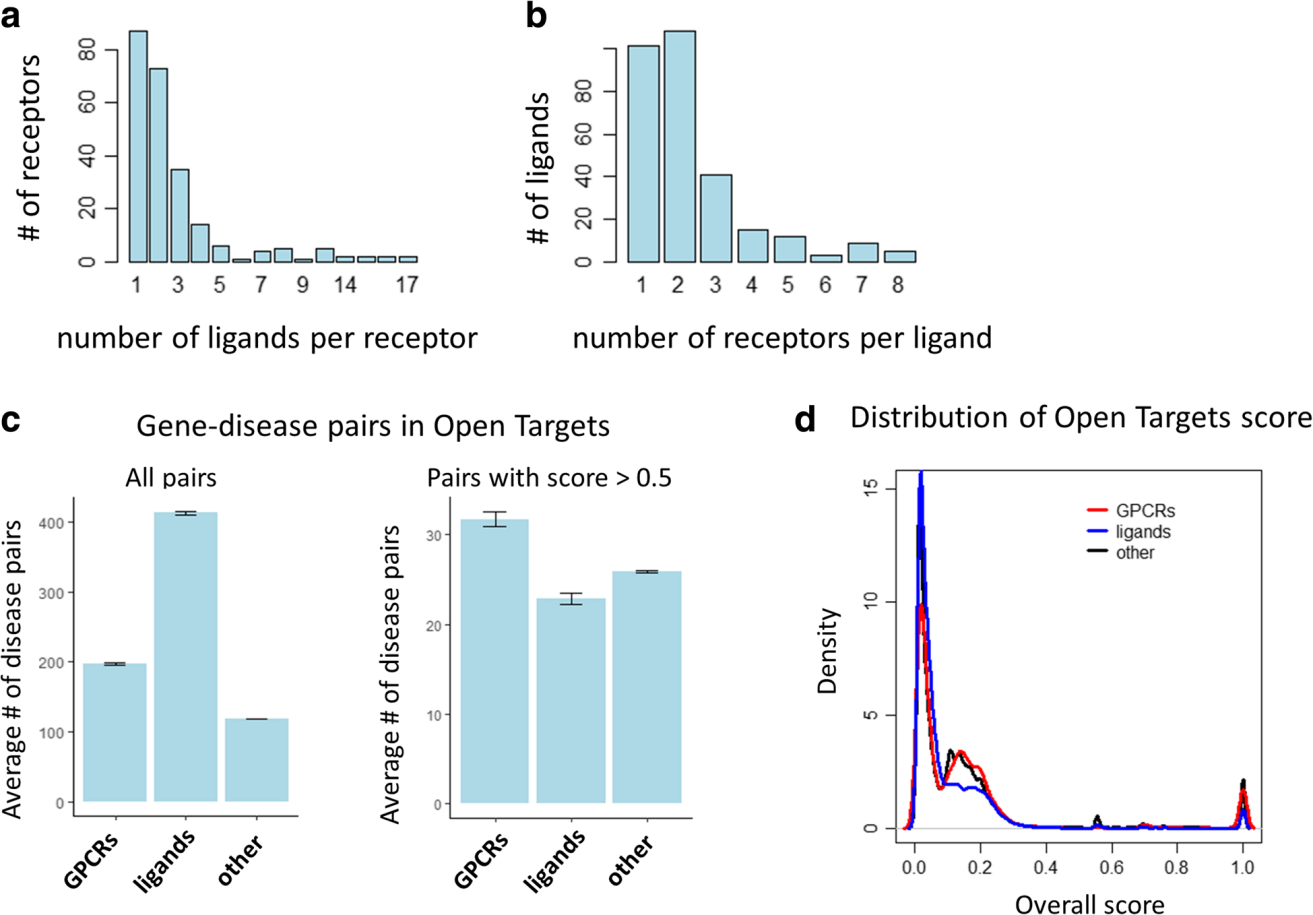
Characterizing GPCRs and endogenous ligands. **a** Number of endogenous ligands per GPCR and (**b**) number of GPCRs per endogenous ligand. **c** Average number of gene-disease pairs by GPCR, endogenous ligand, and all other target types using all pairs (left) and pairs with overall score > 0.5 (right). **d** Distribution of overall scores by target type (GPCR, endogenous ligand, and all other)

We obtained a list of 403 human G-protein coupled receptors (GPCRs) from IUPHAR [19] of which 397 mapped to unique Entrez gene identifiers. In addition, from the same source, we obtained a list of 529 human endogenous ligands [19] which mapped to 412 unique Entrez gene identifiers. It should be noted that some genes encode multiple different peptides (e.g. the GCG gene encodes glucagon, GLP-1, and GLP-2). 119 of the GPCRs and 127 of the endogenous ligands are known to interact according to IUPHAR [19] forming 681 unique receptor-ligand pairs at the gene level. Of these pairs, 34 are 1:1 relationships meaning a GPCR binds exactly one endogenous ligand and vice versa. The remaining GPCR-endogenous ligand pairs are comprised of GPCRs which have up to 17 ligands (Fig. 2a) and ligands that interact with up to 8 different GPCRs (Fig. 2b).

Both GPCRs and endogenous ligands have a considerably higher number of associated disease terms in Open Targets than other classes of genes. The average number of diseases associated with GPCRs is 198 and 413 for endogenous ligands while the average number of associated diseases for all other genes is 119 which is statistically significantly lower (*p* = 1.8 × 10^− 34^ and *p* = 5.5 × 10^− 117^, respectively, Wilcoxon rank sum test). Interestingly, the number of associated disease terms is actually lower than expected for endogenous ligands when we use the relatively stringent 0.5 threshold for the overall Open Targets score but remains higher than expected for GPCRs (23 and 32, respectively, compared to the average of 26) but these comparisons are not significant at the 5% level when using the Wilcoxon rank sum test (*p* = 8.3 × 10^− 2^ and *p* = 8.0 × 10^− 1^, respectively) (Fig. 2c). The increased number of disease associations for GPCRs and endogenous ligands is also reflected in the overall distribution of the Open Targets association score (Fig. 2d).

### Combining GPCR and endogenous ligand disease association evidences

Known GPCR-endogenous ligand pairs can be used to accumulate additional evidence supporting a particular disease hypothesis of interest. For example, the evidence collected in the Open Targets platform suggests that galanin, an endogenous ligand for the GPCR galanin receptor type 2 (GALR2), plays an important role in epilepsy, one of the most common neurological disorders (overall score = 1.0). Indeed, galanin has long been suggested as a potential target to treat epilepsy [20]. In particular, there is evidence found through literature mining (score = 0.004) indicating that galanin depletion from the hippocampus may contribute to the maintenance of seizure activity [21], as well as genetic evidence (score = 1.0) showing that a galanin loss-of-function mutation leads to epilepsy in humans [22]. Interestingly, a recent paper suggests GALR2 as a more suitable potential drug target to treat epilepsy [23], but this literature mining result is currently the only type of evidence supporting the GALR2-epilepsy association in the Open Targets Platform. As a result, the corresponding overall score is a relatively low 0.018 which corresponds to the 20th percentile (Fig. 1a) and by itself does not stand out as a compelling new therapeutic target hypothesis. However, viewing the latter evidence together with the strong genetic evidence for galanin leads to a much stronger hypothesis. As this example illustrates, it may be advantageous to consider ligand-receptor pairs in concert to develop new hypotheses. Additional examples highlighting GPCR-ligand pairs of interest are listed in Table 1. To more systematically identify potential disease indications associated with both an endogenous GPCR ligand and its receptor, we assembled GPCRs and their corresponding endogenous ligands that shared the same disease associations in the Open Targets Platform.

**Table 1:** Examples of known GPCR-endogenous ligand pairs with matching disease indications and corresponding Open Targets overall scores

Disease	GPCR Name	Score	Endogenous ligand Name	Score
Epilepsy	GALR2	0.02	GAL	1.00
Obesity	MC1R	0.04	POMC	1.00
Alzheimer’s disease	FPR2	0.04	APP	1.00
Inflammatory bowel disease	CCR3	0.05	CCL7	0.87
Hypertension	AGTR2	0.15	AGT	1.00
Rheumatoid arthritis	CCR6	0.99	CCL20	0.06
Macular degeneration	CX3CR1	1.00	CX3CL1	0.05
Biliary dyskinesia	SCTR	1.00	VIP	0.02
osteoporosis	CALCR	1.00	ADM	0.04
vascular disease	EDNRA	1.00	EDN2	0.04

**Fig. 3:**
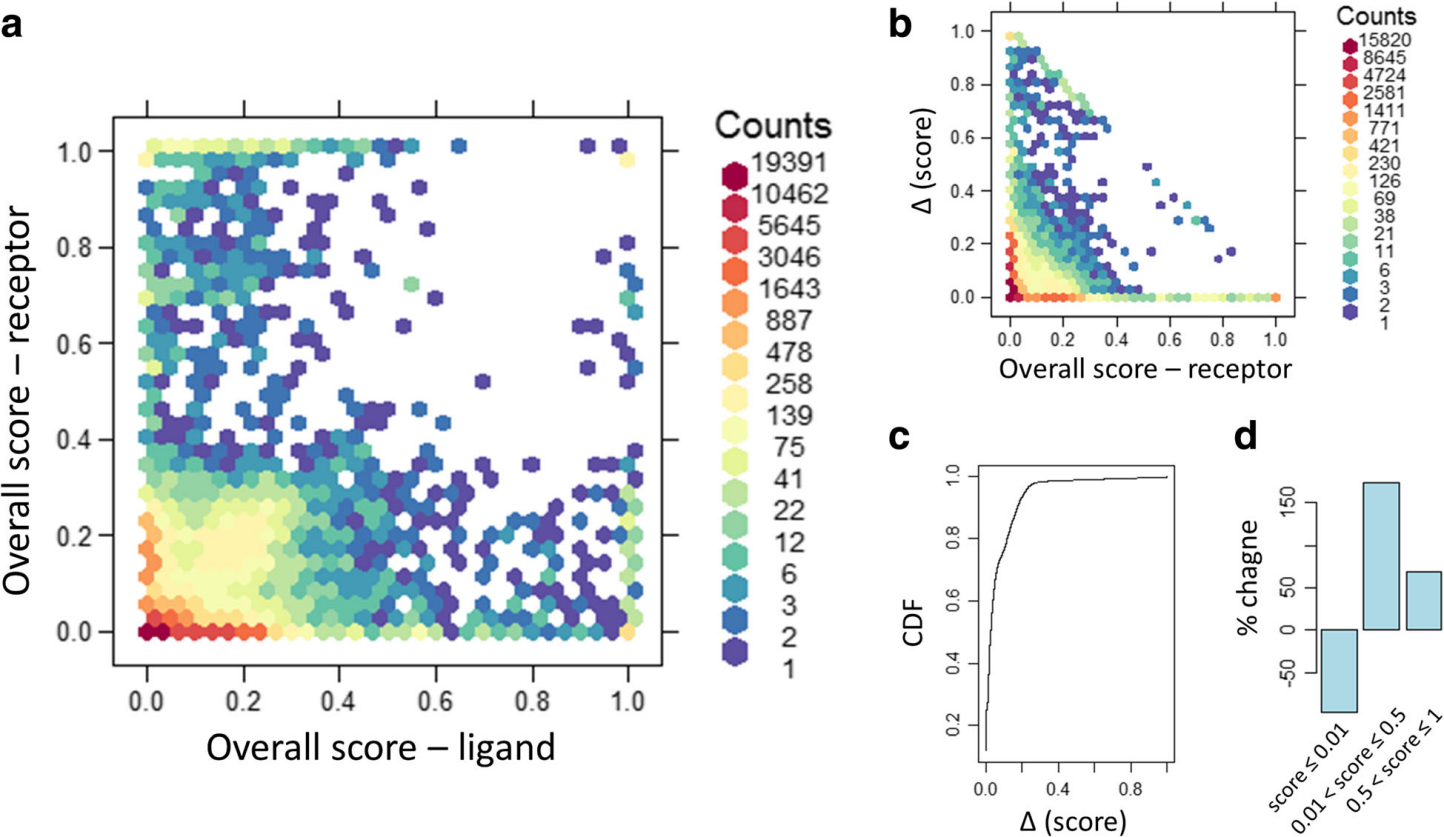
Comparing the overall Open Targets score for disease-GPCR pairs and the corresponding disease-endogenous ligand pairs showing a two dimensional histogram (**a**), the distribution of the increase in overall score comparing the disease-GPCR pairs to the corresponding disease-GPCR/ligand pairs (**b**), the cumulative density function (CDF) for this change in score (**c**), and % change of the number of pairs in the indicated brackets when comparing disease-GPCR/ligand pairs to the corresponding disease-GPCR pair alone (**d)**

Figure 3a and the corresponding Additional file 1: Table S1 shows the overall Open Targets score for disease-GPCR pairs plotted against the score for corresponding disease-endogenous ligand pairs. For this analysis, pairs without any evidence were assigned a score of 0. If there was a strong correlation between disease-GPCR pairs and pairs of the same disease and corresponding endogenous ligand, we would expect most disease-gene pairs in this plot scatter around the diagonal. However, the observed correlation is relatively low (Pearson correlation = 0.21). Figure 3a indicates that there is a large number of GPCR-endogenous ligand pairs where the evidence is strong (e.g. overall score > 0.5) for one but not the other partner, that is, evidence for disease involvement is often asymmetrically reported for one or other partner in these ligand-receptor pairs. It is possible that the involvement in the disease is not mediated through the partner interaction in such cases. However, since the identities of both partners in these interactions are well established, we should consider the evidence for the GPCR and its known endogenous ligand together as a pair to increase our confidence and supportive evidence for potential new target hypotheses. For example, genetic evidence for a disease association with an endogenous ligand may exist but the corresponding GPCR may turn out to be the better drug target due to, for example, druggability. To further quantify the added benefit of combining supportive evidence from GPCRs and ligands we first determined all disease-GPCR pairs and corresponding disease-ligand pairs with positive overall scores. If a pair had a positive score in only one category, we added the corresponding pair in the other category with 0 score. We then created joint disease-GPCR/ligand pairs and assigned a new overall score as the maximum of the scores from the disease-GPCR pairs and corresponding disease-ligand pairs. Figure 3b shows the distribution of the increase in overall score comparing the disease-GPCR pairs to the corresponding disease-GPCR/ligand pairs and Fig. 3c shows cumulative density function (CDF) for this change in score. While 93% of scores increased by 0.2 or less, many new high confidence pairs also emerged: 648 disease-GPCR/ligand pairs had a score of 0.5 or higher but did not have any supportive evidence (i.e. score = 0) for the corresponding disease-GPCR pair alone without considering the ligand. Of those, 355 pairs had a new score of 1.0 compared to the previous score of 0. Comparing the number of disease-GPCR/ligand pairs to the number of corresponding disease-GPCR pairs alone, the number of pairs without any evidence (i.e. score = 0) decreased by 62% and the number of high-confidence pairs (score > 0.5) was more than 1100 higher, a 69% increase (Fig. 3d).

### GPCRs and endogenous peptidergic ligands and the highest stage in global pharma pipelines

**Fig. 4:**
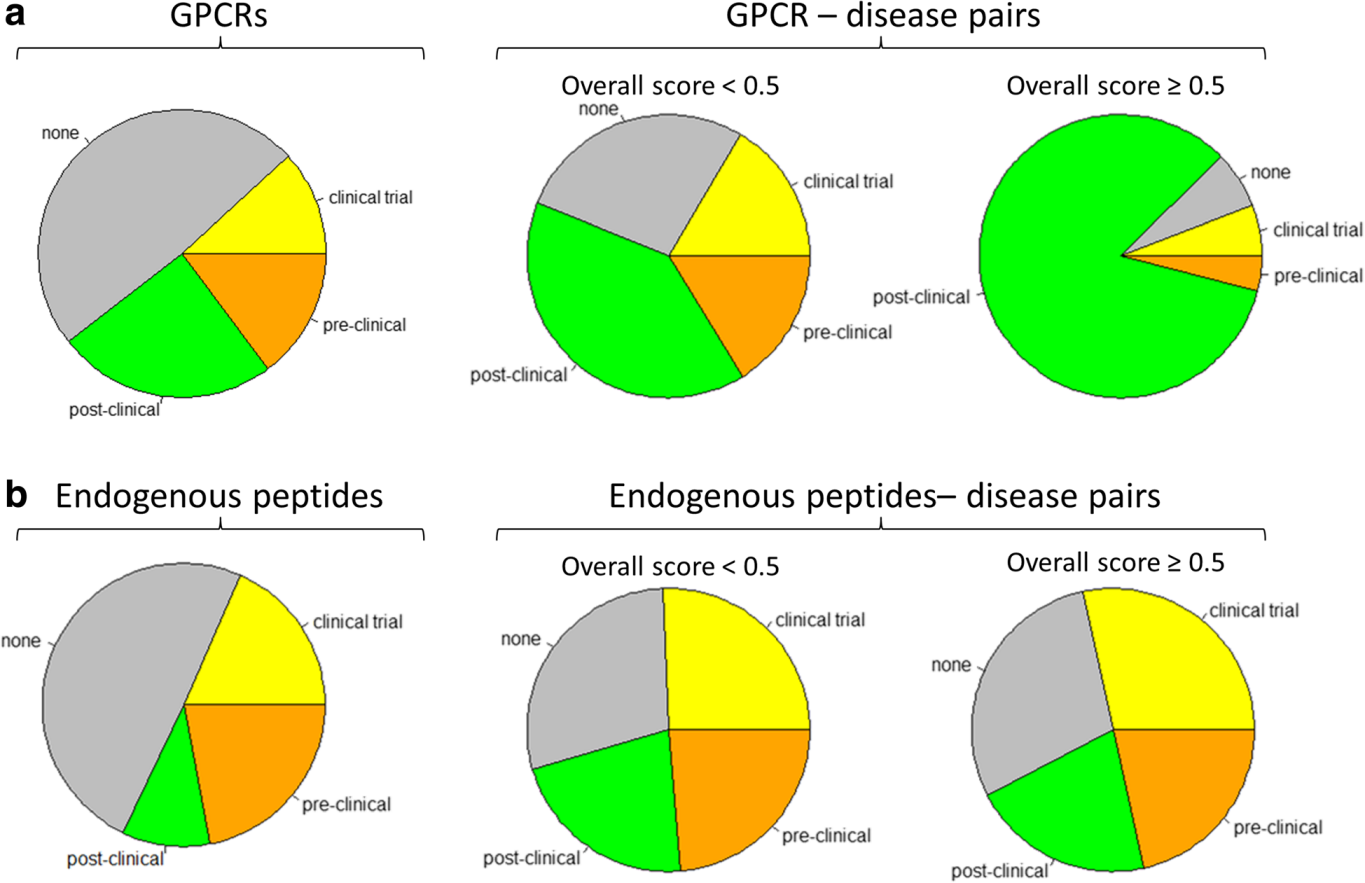
GPCRs (**a**) and endogenous peptidergic ligands (**b**) and the highest stage in global pharma pipeline. In each panel, the leftmost chart shows the distribution of highest stage (post-clinical, clinical trial, pre-clinical, none) by target type while the other two charts show such distribution among the gene-disease pairs within the Open Targets platform stratified by corresponding overall score (< 0.5, middle; ≥0.5 right)

Based on the past success of GPCRs as drug targets [2], GPCRs that have disease associations with a high score in the Open Targets Platform but are not currently pursued by the industry may potentially be high priority targets for the development of new therapies. Conversely, GPCRs with existing drug discovery programs as well as high scoring disease associations provide potential drug repurposing opportunities for compounds modulating these GPCRs if the top-ranked disease derived from Open Targets is different from the current indication pursued. To more closely examine this approach, we obtained a database of current drug discovery programs [24] and determined the highest stage in the drug discovery pipeline for each GPCR and endogenous peptide. Approximately half of the previously uniquely identified GPCRs and endogenous peptides had at least one program in the drug discovery pipeline (203 out of 397, and 209 out of 412, respectively; Fig. 4). We then stratified GPCR-disease pairs and endogenous peptide-disease pairs by the highest pipeline stage of the corresponding GPCR and peptide, respectively. Approximately 73% of GPCR-disease pairs with an overall Open Targets score below 0.5 had at least one program in the drug discovery pipeline for that target and this number increased to 93% for the GPCR-disease pairs with an overall Open Targets score of 0.5 or higher which corresponds to the 95th percentile of the overall score distribution as described above. In nearly 84% of such pairs, the GPCR has been recorded in at least one post-clinical stage of the drug discovery pipeline and only 6.5% of such pairs involve GPCRs without any drug discovery program (Fig. 4). Together, 56% of the GPCR-disease pairs involved GPCRs that had reached a clinical stage and those pairs had significantly higher overall scores (*p* = 3.8 × 10^− 34^, Wilcoxon rank sum test). Similarly, 47% of the ligand-disease pairs involved endogenous ligands that had reached a clinical stage and those pairs also had significantly higher overall scores (*p* = 1.3 × 10^− 29^, Wilcoxon rank sum test). However, at least some of this relative over-representation of the late-stage pipeline among pairs with overall Open Targets score of 0.5 or higher may be driven by evidence resulting from the very same drug discovery programs as individual evidence types contribute differently to this enrichment. For example, we observed that genetic association and animal model evidence appears to be independent of pipeline status while literature evidence does not. For endogenous peptides, the differences between lower Open Targets scores (< 0.5) and high Open Targets scores (≥0.5) are less prominent. For example, the endogenous peptide-disease pairs with an overall Open Targets score below 0.5 as well as the pairs with a score of 0.5 or higher both included approximately 29% of pairs where the ligand did not have any program in the drug discovery pipeline.

To illustrate how the Open Targets platform might be applied to prioritize a particular target-disease hypothesis for drug discovery, consider one of the examples listed in Table 2, GPR35 for inflammatory bowel diseases (IBD). The incidence and prevalence of IBD such as Crohn’s disease and ulcerative colitis are increasing over time globally [25] and estimates suggest that ~ 1.4 million people in the United States and 250,000 people in the United Kingdom suffer from this disease [25, 26]. The aetiology is currently not well known, and it is hypothesized that the genetically susceptible host suffers from compromised intestinal immune system response to commensal bacteria [25]. Currently, there is no known cure for this chronic condition, and constant care & symptomatic treatment is needed for patients suffering with this condition. Several genome-wide association studies have identified the GPR35 locus as one of the susceptibility loci for IBD [27, 28]. As the evidence listed in the Open Targets platform shows, GPR35 is currently investigated in clinical trials for pruritus and mastocytosis and presents a promising new therapeutic target for a number of disease indications including inflammatory and cardiovascular disease [29–33]. Currently, no drug targeting GPR35 is approved for IBD. Taken together, the evidence compiled in Open Targets strongly suggests that GPR35 could be investigated as a novel therapeutic option for IBD. It should be noted that lodoxamide, a GPR35 agonist, is an approved drug for conjunctivitis which could potentially be repositioned for IBD.

**Table 2:** Examples of potential disease indications for GPCRs and endogenous peptides and corresponding Open Targets overall scores that represent new target hypotheses or potential repurposing opportunities

		Gene	Disease term	Score
GPCRs	Repurposing opportunity	GPR35	Inflammatory bowel disease	1.00
		CXCR4	Infectious disease	1.00
		PTGER4	Inflammatory bowel disease	0.90
		TSHR	Graves disease	0.67
	New target	NPSR1	Asthma	1.00
		LPAR6	Alopecia	1.00
		CELSR2	Coronary heart disease	0.92
		GPR65	Crohn’s disease	0.81
Endogenous peptide	Repurposing opportunity	TNFSF15	Inflammatory bowel disease	1.00
		AGT	Hypertension	1.00
		IL17F	Immune system disease	1.00
		CALCA	Cardiovascular disease	1.00
	New target	FBN1	Vascular disease	1.00
		COL3A1	Cardiovascular disease	1.00
		NPPA	Cardiomyopathy	1.00
		IL33	Respiratory system disease	0.77

Another such example includes C C-X-C motif chemokine receptor 4 (CXCR4) as a potential new drug target for infectious diseases. The Open Targets platform identifies weak supporting evidence from RNA expression and genetic associations (score = 0.01 in each case) but strong pathway evidence. CXCR4 is part of the ‘Binding and entry of HIV virion’ pathway, a manually curated pathway from Reactome (score = 1.0), but is also listed in various relevant gene ontology pathways such as GO:0001618 ‘virus receptor activity’ which can easily be determined by following the link-out to the Uniprot database within the Open Target platform entry for CXCR4. The CXCR4 receptor is actually well known to play a critical role for the entry of the human immunodeficiency virus (HIV) into CD4+ T-cells but other viruses use this entry as well [34]. The literature text mining evidence shown in the Open Targets platform receives a score of 0.21. However, the platform identifies nearly 1200 publications further strengthening the hypothesis. Table 2 lists additional specific examples of possible repurposing opportunities and Additional file 1: Table S2 lists examples with overall score of 0.5 or higher.

## Discussion

Drug discovery and development programs focus on achieving therapeutic efficacy upon modulation of a specific drug target with a molecule in a patient population. A number of computational approaches to aid in target identification have been considered, and along with various bioinformatics resources they also point to the application of cheminformatics based approaches for ligand discovery [35]. However, the hypothesis that modulation of a specific target may potentially result in therapeutic benefit is often based upon years of scientific work which involves generating and/or accumulating experimental evidence in an iterative manner and then meaningfully integrating that information to further lend support to that hypothesis. The scientific evidence to build that hypothesis comes from multiple sources; and integration as well as evaluation of that data is critical for drug discovery programs. Especially in a world of rapidly growing data, the ability to integrate data from multiple sources with platforms such as Open Targets, presents an opportunity to systematically evaluate the available evidence to quickly generate hypotheses to identify targets that may further be followed up with additional experimentation [13]. It should be noted that the purpose of such efforts is not necessarily to identify novel target-disease associations per se but rather to prioritize such associations in order to identify the most promising opportunities for drug discovery.

In this study, we present the development and application of a systematic target identification approach on data from Open Targets platform. We first examine and characterize the distribution of the Open Targets score and its relationship with the individual evidence type scores. We then focus on a very successful target class of proteins in drug discovery, G-protein coupled receptors (GPCRs), along with their endogenous ligands. Specifically, we use a list of GPCRs and endogenous peptidergic ligands from IUPHAR, map them to Entrez gene identifiers, assemble data from various sources of evidence in the Open Targets platform and associate the disease terms with therapy areas for broader categorization. Although we are focusing on GPCR-ligand pairs in our analysis, our approach can be generalized to any heteromultimeric proteins or potentially to any pairs of proteins known to directly interact. Finally, we compare the Open Targets derived target-indication hypotheses (based on gene-disease associations) to the global pharmaceutical drug discovery landscape as a means to evaluate some of these hypotheses.

We observed that both GPCRs and endogenous ligands have a higher than expected number of associated disease terms in Open Targets. One explanation for this seemingly higher disease-relevance could be that these classes of proteins simply are better studied and understood than the proteome as a whole due to the extraordinary success of these protein classes as therapeutic drug targets [36]. The relatively high number of GPCR-disease pairs with an existing drug discovery program seems to confirm this view and suggests that this class of potential drug targets is well suited to evaluate the Open Targets score. We found that an Open Targets score of 0.5 corresponds to the 95th percentile of the overall score distribution and that over 90% of GPCR-disease pairs with a score of 0.5 or higher had a corresponding drug discovery program for that GPCR. This suggests that an overall Open Targets score of 0.5 could be used as a high confidence threshold when evaluating potential new target-indication hypotheses. We also found that confidence in such hypotheses was increased by more individual supporting evidence types.

Our current study highlights the benefit of combining different, independent sources of evidence supporting a target-disease hypothesis to increase confidence in its validity. This relationship was intentionally reflected in the design of the individual scores and, also in the overall score [13]. In particular, the overall score increases with the number of positive individual scores for a given target-disease hypothesis as shown in Fig. 2c. Another way the Open Targets platform can be used to accumulate existing supporting evidence is by combining data for closely related targets such as through a shared molecular pathway, heteromultimeric protein complexes, or through receptor-ligand pairs such as the examples highlighted in Table 1. It should be noted that the interpretation of an observed association between genes or proteins and diseases or medical conditions is not trivial. Such relationship may or may not be causal and it may be direct or indirect. Furthermore, it is often unclear if the disease association is due to an increase or decrease in activity or abundance of the functional protein. Some of the evidence integrated in the Open Targets database provides more clarity in this regard (e.g. availability of a known drug, Mendelian trait, knock-out animal model). In the context of GPCR-ligand relationships, it is also important to consider whether a ligand acts in a pathological or therapeutic role. For example, glucagon-like peptide-1 (GLP-1) can decrease blood sugar levels which has led to the development of GLP-1 receptor agonists as new drugs to treat type 2 diabetes [37]. Conversely, vasopressin plays a central role in the pathogenesis of hyponatremia which has led to the development of vasopressin receptor antagonists as a treatment [38]. As a result, each target-disease association of interest requires further careful evaluation of the evidence and subsequent experimental validation.

As with any systematic or global computational solution to a biological or biomedical problem, simplifications and generalizations are required. Therefore, a general approach applied to all disease terms and all potential drug targets such as the Open Targets platform may be more suitable in some situations than in others. For example, the current evidence presented by the Open Targets platform concentrates on data generated through methods that focus on the DNA or RNA level but the action of a therapeutic drug is most often mediated at the protein level, e.g. by disrupting protein-protein interactions [39] or protein complexes comprised of multiple different genes [40]. In other cases, a protein might have multiple splice forms, or both a membrane-bound and a soluble form. In addition, in some cases the same gene encodes multiple different peptides such as the GCG gene encoding glucagon, GLP-1, and GLP-2, each of which may have different receptors as well as different disease associations. Additional evidence that reflects such complexities could enhance the utility of the platform.

It should also be noted that as with any computational approach, false positive and false negative results are unavoidable and should be expected. Each target-disease pair merely represents a hypothesis that serves as a starting point for drug discovery scientists looking to begin a new research program. These hypotheses still require careful evaluation, prioritization, and experimental validation. Two final examples illustrate this point. Neuropeptide S receptor 1 (NPSR1) was identified as a potential new drug target for asthma in the Open Targets platform. Strong genetic evidence supports the hypothesis [41–43] and the Open Targets platform identifies over 60 publications suggesting a role of NPSR1 in asthma but the exact mechanism of NPSR1 in the disease remains elusive. Although increased NPSR1 protein levels in plasma were reported in asthma [44] and increased NPSR1 mRNA expression was observed in eosinophils from severe asthmatic patients [45], experiments in an experimental asthma mouse model showed no impact of Npsr1 deletion on airway inflammation or hyper-responsiveness, and the authors suggested that NPSR1 affects the disease through a central nervous system-mediated pathway [46]. Similarly, G protein-coupled receptor 65 (GPR65), a receptor for psychosine and several related glycosphingolipids, received a strong Open Targets score for Crohn’s disease mostly due to its strong genetic association [28, 47, 48]. The protein’s role in the disease is not entirely clear but it may play role in proton sensing [49] or acid sensing [50, 51] and may regulate cytokine production of T cells and macrophages [52, 53]. These examples further illustrate the importance of systematically mining the Open Targets data and then prioritizing target-indication pairs for follow-up experimental work to validate the hypotheses.

## Conclusion

In summary, by utilizing the Open Targets platform, data, and evidence model, and by interrogation of underlying and additional data, we have been able to generate various GPCR – indication pair combinations, which form the basis for development hypotheses for potential drug discovery programs and this approach can be generalized in a straightforward fashion to include other drug target classes.

## Methods

### Open targets platform data

Open Targets gene-disease pairs and scores (September 9, 2017 version, Release 3.2; JSON format) were downloaded from the Open Targets website [13]. The data download was parsed capturing disease term and Experimental Factor Ontology (EFO) identifier, Ensembl gene identifier and symbol, as well as 8 scores: overall, genetic association, somatic mutation, known drug RNA expression, affected pathway, animal model, and literature mining scores, respectively. Ensembl gene identifiers were mapped to Entrez genes and official HUGO gene symbols using relevant Bioconductor packages [54, 55].

### Experimental factor ontology (EFO)

The EFO [56] was downloaded in OBO format (September 7, 2017 version). The ontology was parsed recursively using the “is_a” relationships encoded in each entry in order to determine one or more therapeutic areas for each disease term. Specifically, an EFO term was considered a therapeutic area if it was a directly associated with “disease” (EFO:0000408) through an “is_a” relationship. A small number of such top-level terms were manually remapped to a different therapeutic area (e.g. “heavy metal poisoning”, “malignant epitheloid mesothelioma”, and “sudden infant death syndrome”).

### GPCRs and endogenous peptides

Three data tables were downloaded from IUPHAR (http://www.guidetopharmacology.org) [19]: (a) a list of GPCRs, (b) a list of endogenous peptides, and (c) the list of all interaction data for endogenous ligands and their GPCR targets. GPCRs were mapped to Entrez gene identifiers by gene symbols, and endogenous peptides were mapped to Entrez gene identifiers by Uniprot IDs using relevant Bioconductor packages [54, 55] in both cases.

### Comparison to Pharmapipeline

The Pharmapipeline database was retrieved from Informa PLC [24]. It contains data on the current global pharmaceutical drug discovery pipeline and identifies drugs discovery programs, their current status, molecular target, and indication, among other data. Molecular target identifiers were mapped to one or more Entrez gene IDs and drugs without a matching gene identifier were removed from further analyses. We summarized the drug discovery pipeline stages as follows: none (“N/A”, global status 1), pre-clinical (global status 2–5), clinical trial (global status 7–9), and post-clinical (global status 10–13). For target-indication pairs with multiple corresponding drug discovery programs, we chose the highest stage as representative.

## Additional file

Additional file 1: **Table S1.** Overall Open Targets score for disease-GPCR pairs and the corresponding disease-endogenous ligand pairs. **Table S2.** Possible repurposing opportunities with overall Open Targets score of 0.5 or higher. (XLSX 1930 kb)

## Abbreviations

CDF: Cumulative density function
CXCR4: C-X-C chemokine receptor type 4
EFO: Experimental Factor Ontology
FDA: United States Food and Drug Administration
GALR2: Galanin receptor type 2
GCG: Glucagon
GLP-1: Glucagon-like peptide 1
GLP-2: Glucagon-like peptide 2
GPCR: G-protein-coupled receptor
GPR35: G protein-coupled receptor 35
GPR65: G protein-coupled receptor 65
HIV: Human immunodeficiency virus
HUGO: Human Genome Organisation
IBD: Inflammatory bowel disease
NPSR1: Neuropeptide S receptor 1
RNA: Ribonucleic acid

## References

[1] Oprea TI, Bologa CG, Brunak S, Campbell A, Gan GN, Gaulton A, Gomez SM, Guha R, Hersey A, Holmes J (2018). Unexplored therapeutic opportunities in the human genome. Nat Rev Drug Discov.

[2] Rask-Andersen M, Masuram S, Schioth HB (2014). The druggable genome: evaluation of drug targets in clinical trials suggests major shifts in molecular class and indication. Annu Rev Pharmacol Toxicol.

[3] Lu S, Zhang J. Small molecule allosteric modulators of G-protein-coupled receptors: drug-target interactions. J Med Chem. 2018. 10.1021/acs.jmedchem.7b01844 29457894

[4] Topiol S (1705). Current and future challenges in GPCR drug discovery. Methods Mol Biol.

[5] Hauser AS, Attwood MM, Rask-Andersen M, Schioth HB, Gloriam DE (2017). Trends in GPCR drug discovery: new agents, targets and indications. Nat Rev Drug Discov.

[6] Fosgerau K, Hoffmann T (2015). Peptide therapeutics: current status and future directions. Drug Discov Today.

[7] Vass M, Kooistra AJ, Yang D, Stevens RC, Wang MW, de Graaf C (2018). Chemical diversity in the G protein-coupled receptor superfamily. Trends Pharmacol Sci.

[8] Pandy-Szekeres G, Munk C, Tsonkov TM, Mordalski S, Harpsoe K, Hauser AS, Bojarski AJ, Gloriam DE (2018). GPCRdb in 2018: adding GPCR structure models and ligands. Nucleic Acids Res.

[9] Nguyen DT, Mathias S, Bologa C, Brunak S, Fernandez N, Gaulton A, Hersey A, Holmes J, Jensen LJ, Karlsson A (2017). Pharos: collating protein information to shed light on the druggable genome. Nucleic Acids Res.

[10] Pinero J, Bravo A, Queralt-Rosinach N, Gutierrez-Sacristan A, Deu-Pons J, Centeno E, Garcia-Garcia J, Sanz F, Furlong LI (2017). DisGeNET: a comprehensive platform integrating information on human disease-associated genes and variants. Nucleic Acids Res.

[11] Mungall CJ, McMurry JA, Kohler S, Balhoff JP, Borromeo C, Brush M, Carbon S, Conlin T, Dunn N, Engelstad M (2017). The monarch initiative: an integrative data and analytic platform connecting phenotypes to genotypes across species. Nucleic Acids Res.

[12] Pletscher-Frankild S, Palleja A, Tsafou K, Binder JX, Jensen LJ (2015). DISEASES: text mining and data integration of disease-gene associations. Methods.

[13] Koscielny G, An P, Carvalho-Silva D, Cham JA, Fumis L, Gasparyan R, Hasan S, Karamanis N, Maguire M, Papa E (2017). Open targets: a platform for therapeutic target identification and validation. Nucleic Acids Res.

[14] Butte AJ, Kohane IS. Mutual information relevance networks: functional genomic clustering using pairwise entropy measurements. Pac Symp Biocomput. 2000:418–29. 10.1142/9789814447331_0040 10902190

[15] Ferrero E, Dunham I, Sanseau P (2017). In silico prediction of novel therapeutic targets using gene-disease association data. J Transl Med.

[16] Bartfai T, Lees GV (2013). The future of drug discovery: who decides which diseases to treat?: academic press.

[17] Finan Chris, Gaulton Anna, Kruger Felix A., Lumbers R. Thomas, Shah Tina, Engmann Jorgen, Galver Luana, Kelley Ryan, Karlsson Anneli, Santos Rita, Overington John P., Hingorani Aroon D., Casas Juan P. (2017). The druggable genome and support for target identification and validation in drug development. Science Translational Medicine.

[18] Kafkas S, Dunham I, McEntyre J (2017). Literature evidence in open targets - a target validation platform. J Biomed Semantics.

[19] Southan C, Sharman JL, Benson HE, Faccenda E, Pawson AJ, Alexander SP, Buneman OP, Davenport AP, McGrath JC, Peters JA (2016). The IUPHAR/BPS guide to PHARMACOLOGY in 2016: towards curated quantitative interactions between 1300 protein targets and 6000 ligands. Nucleic Acids Res.

[20] Mazarati A, Langel U, Bartfai T (2001). Galanin: an endogenous anticonvulsant?. Neuroscientist.

[21] Clynen E, Swijsen A, Raijmakers M, Hoogland G, Rigo JM (2014). Neuropeptides as targets for the development of anticonvulsant drugs. Mol Neurobiol.

[22] Guipponi M, Chentouf A, Webling KE, Freimann K, Crespel A, Nobile C, Lemke JR, Hansen J, Dorn T, Lesca G (2015). Galanin pathogenic mutations in temporal lobe epilepsy. Hum Mol Genet.

[23] Hui WQ, Cheng Q, Liu TY, Ouyang Q (2016). Homology modeling, docking, and molecular dynamics simulation of the receptor GALR2 and its interactions with galanin and a positive allosteric modulator. J Mol Model.

[24] Informa Pharmaprojects [https://pharmaintelligence.informa.com/products-and-services/data-and-analysis/pharmaprojects].

[25] Molodecky NA, Soon IS, Rabi DM, Ghali WA, Ferris M, Chernoff G, Benchimol EI, Panaccione R, Ghosh S, Barkema HW (2012). Increasing incidence and prevalence of the inflammatory bowel diseases with time, based on systematic review. Gastroenterol.

[26] Ng SC, Tang W, Ching JY, Wong M, Chow CM, Hui AJ, Wong TC, Leung VK, Tsang SW, Yu HH (2013). Incidence and phenotype of inflammatory bowel disease based on results from the Asia-pacific Crohn's and colitis epidemiology study. Gastroenterol.

[27] Anderson CA, Boucher G, Lees CW, Franke A, D'Amato M, Taylor KD, Lee JC, Goyette P, Imielinski M, Latiano A (2011). Meta-analysis identifies 29 additional ulcerative colitis risk loci, increasing the number of confirmed associations to 47. Nat Genet.

[28] Liu JZ, van Sommeren S, Huang H, Ng SC, Alberts R, Takahashi A, Ripke S, Lee JC, Jostins L, Shah T (2015). Association analyses identify 38 susceptibility loci for inflammatory bowel disease and highlight shared genetic risk across populations. Nat Genet.

[29] Divorty N, Mackenzie AE, Nicklin SA, Milligan G (2015). G protein-coupled receptor 35: an emerging target in inflammatory and cardiovascular disease. Front Pharmacol.

[30] Heynen-Genel S, Dahl R, Shi S, Sauer M, Hariharan S, Sergienko E, Dad S, Chung TDY, Stonich D, Su Y et al: Selective GPR35 Antagonists - Probes 1 & 2. In: Probe Reports from the NIH Molecular Libraries Program. Bethesda (MD); 2010.

[31] Mackenzie AE, Lappin JE, Taylor DL, Nicklin SA, Milligan G (2011). GPR35 as a novel therapeutic target. Front Endocrinol (Lausanne).

[32] Maravillas-Montero JL, Burkhardt AM, Hevezi PA, Carnevale CD, Smit MJ, Zlotnik A (2015). Cutting edge: GPR35/CXCR8 is the receptor of the mucosal chemokine CXCL17. J Immunol.

[33] Shore DM, Reggio PH (2015). The therapeutic potential of orphan GPCRs, GPR35 and GPR55. Front Pharmacol.

[34] Arnolds KL, Spencer JV (2014). CXCR4: a virus's best friend?. Infect Genet Evol.

[35] Katsila T, Spyroulias GA, Patrinos GP, Matsoukas MT (2016). Computational approaches in target identification and drug discovery. Comput Struct Biotechnol J.

[36] Santos R, Ursu O, Gaulton A, Bento AP, Donadi RS, Bologa CG, Karlsson A, Al-Lazikani B, Hersey A, Oprea TI (2017). A comprehensive map of molecular drug targets. Nat Rev Drug Discov.

[37] Scott RA, Freitag DF, Li L, Chu AY, Surendran P, Young R, Grarup N, Stancakova A, Chen Y, Varga TV (2016). A genomic approach to therapeutic target validation identifies a glucose-lowering GLP1R variant protective for coronary heart disease. Sci Transl Med.

[38] Rondon-Berrios H, Berl T (2016). Vasopressin receptor antagonists: characteristics and clinical role. Best Pract Res Clin Endocrinol Metab.

[39] Chene P (2006). Drugs targeting protein-protein interactions. ChemMedChem.

[40] Bibo-Verdugo B, Jiang Z, Caffrey CR, O'Donoghue AJ (2017). Targeting proteasomes in infectious organisms to combat disease. FEBS J.

[41] Acevedo N, Ezer S, Kebede Merid S, Gaertner VD, Soderhall C, D'Amato M, Kabesch M, Melen E, Kere J, Pulkkinen V (2017). Neuropeptide S (NPS) variants modify the signaling and risk effects of NPS receptor 1 (NPSR1) variants in asthma. PLoS One.

[42] Kormann MS, Carr D, Klopp N, Illig T, Leupold W, Fritzsch C, Weiland SK, von Mutius E, Kabesch M (2005). G-protein-coupled receptor polymorphisms are associated with asthma in a large German population. Am J Respir Crit Care Med.

[43] Melen E, Bruce S, Doekes G, Kabesch M, Laitinen T, Lauener R, Lindgren CM, Riedler J, Scheynius A, van Hage-Hamsten M (2005). Haplotypes of G protein-coupled receptor 154 are associated with childhood allergy and asthma. Am J Respir Crit Care Med.

[44] Hamsten C, Haggmark A, Grundstrom J, Mikus M, Lindskog C, Konradsen JR, Eklund A, Pershagen G, Wickman M, Grunewald J (2016). Protein profiles of CCL5, HPGDS, and NPSR1 in plasma reveal association with childhood asthma. Allergy.

[45] Ilmarinen P, James A, Moilanen E, Pulkkinen V, Daham K, Saarelainen S, Laitinen T, Dahlen SE, Kere J, Dahlen B (2014). Enhanced expression of neuropeptide S (NPS) receptor in eosinophils from severe asthmatics and subjects with total IgE above 100IU/ml. Peptides.

[46] Zhu H, Perkins C, Mingler MK, Finkelman FD, Rothenberg ME (2011). The role of neuropeptide S and neuropeptide S receptor 1 in regulation of respiratory function in mice. Peptides.

[47] Franke A, McGovern DP, Barrett JC, Wang K, Radford-Smith GL, Ahmad T, Lees CW, Balschun T, Lee J, Roberts R (2010). Genome-wide meta-analysis increases to 71 the number of confirmed Crohn's disease susceptibility loci. Nat Genet.

[48] de Lange KM, Moutsianas L, Lee JC, Lamb CA, Luo Y, Kennedy NA, Jostins L, Rice DL, Gutierrez-Achury J, Ji SG (2017). Genome-wide association study implicates immune activation of multiple integrin genes in inflammatory bowel disease. Nat Genet.

[49] Wang JQ, Kon J, Mogi C, Tobo M, Damirin A, Sato K, Komachi M, Malchinkhuu E, Murata N, Kimura T (2004). TDAG8 is a proton-sensing and psychosine-sensitive G-protein-coupled receptor. J Biol Chem.

[50] Ishii S, Kihara Y, Shimizu T (2005). Identification of T cell death-associated gene 8 (TDAG8) as a novel acid sensing G-protein-coupled receptor. J Biol Chem.

[51] Ihara Y, Kihara Y, Hamano F, Yanagida K, Morishita Y, Kunita A, Yamori T, Fukayama M, Aburatani H, Shimizu T (2010). The G protein-coupled receptor T-cell death-associated gene 8 (TDAG8) facilitates tumor development by serving as an extracellular pH sensor. Proc Natl Acad Sci U S A.

[52] Onozawa Y, Fujita Y, Kuwabara H, Nagasaki M, Komai T, Oda T (2012). Activation of T cell death-associated gene 8 regulates the cytokine production of T cells and macrophages in vitro. Eur J Pharmacol.

[53] Mogi C, Tobo M, Tomura H, Murata N, He XD, Sato K, Kimura T, Ishizuka T, Sasaki T, Sato T (2009). Involvement of proton-sensing TDAG8 in extracellular acidification-induced inhibition of proinflammatory cytokine production in peritoneal macrophages. J Immunol.

[54] Carlson M: org.Hs.eg.db: Genome wide annotation for Human. In*.*; 2016.

[55] Huber W, Carey VJ, Gentleman R, Anders S, Carlson M, Carvalho BS, Bravo HC, Davis S, Gatto L, Girke T (2015). Orchestrating high-throughput genomic analysis with Bioconductor. Nat Methods.

[56] Malone J, Holloway E, Adamusiak T, Kapushesky M, Zheng J, Kolesnikov N, Zhukova A, Brazma A, Parkinson H (2010). Modeling sample variables with an experimental factor ontology. Bioinformatics.

